# Characteristic and Functional Analysis of a Newly Established Porcine Small Intestinal Epithelial Cell Line

**DOI:** 10.1371/journal.pone.0110916

**Published:** 2014-10-22

**Authors:** Jing Wang, Guangdong Hu, Zhi Lin, Lei He, Lei Xu, Yanming Zhang

**Affiliations:** College of Veterinary Medicine, Northwest A&F University, Yangling, Shaanxi, China; University of Osnabrueck, Germany

## Abstract

The mucosal surface of intestine is continuously exposed to both potential pathogens and beneficial commensal microorganisms. Recent findings suggest that intestinal epithelial cells, which once considered as a simple physical barrier, are a crucial cell lineage necessary for maintaining intestinal immune homeostasis. Therefore, establishing a stable and reliable intestinal epithelial cell line for future research on the mucosal immune system is necessary. In the present study, we established a porcine intestinal epithelial cell line (ZYM-SIEC02) by introducing the human telomerase reverse transcriptase (hTERT) gene into small intestinal epithelial cells derived from a neonatal, unsuckled piglet. Morphological analysis revealed a homogeneous cobblestone-like morphology of the epithelial cell sheets. Ultrastructural indicated the presence of microvilli, tight junctions, and a glandular configuration typical of the small intestine. Furthermore, ZYM-SIEC02 cells expressed epithelial cell-specific markers including cytokeratin 18, pan-cytokeratin, sucrase-isomaltase, E-cadherin and ZO-1. Immortalized ZYM-SIEC02 cells remained diploid and were not transformed. In addition, we also examined the host cell response to *Salmonella* and LPS and verified the enhanced expression of mRNAs encoding IL-8 and TNF-α by infection with *Salmonella enterica serovars Typhimurium* (*S. Typhimurium*). Results showed that IL-8 protein expression were upregulated following Salmonella invasion. TLR4, TLR6 and IL-6 mRNA expression were upregulated following stimulation with LPS, ZYM-SIEC02 cells were hyporeponsive to LPS with respect to IL-8 mRNA expression and secretion. TNFα mRNA levels were significantly decreased after LPS stimulation and TNF-α secretion were not detected challenged with *S. Typhimurium* neither nor LPS. Taken together, these findings demonstrate that ZYM-SIEC02 cells retained the morphological and functional characteristics typical of primary swine intestinal epithelial cells and thus provide a relevant *in vitro* model system for future studies on porcine small intestinal pathogen-host cell interactions.

## Introduction

Pigs of all ages are susceptible to intestinal diseases, which most commonly present as diarrhea [1]. However, piglets are especially vulnerable to infection by bacteria, viruses, parasites and other etiologic agents that cause primary intestinal diseases. Intestinal diseases in piglets have both high morbidity and mortality, which results in large losses in the livestock industry each year. Previous studies have been largely performed in animal infection models [2], however, the study of molecular mechanisms of enteropathogen infections is limited by the availability of reliable and relevant established porcine cell lines.

The intestinal epithelial monolayer acts not only as a physical barrier but also plays a critical role in preventing macromolecules and pathogenic microorganisms in the gut lumen from penetrating to the underlining mucosa [2]. The mucosal surface is continuously exposed to commensal microorganisms and/or innocuous environmental antigens, and the intestinal mucosal immune system is exquisitely sensitive to the challenge of constant immunological stimulation [3]. Many studies have described the host-pathogen interaction in short-term intestinal epithelial cell cultures derived from humans [4]–[7] and from a variety of animals [8], including mice [9]–[11], rats [12]–[15], rabbits [16], and cattle [17]–[21]. Non-transformed long-term swine epithelial cell lines from intestinal sections are available so far, e.g. IPEC-1 from pig ileum and jejunum [22] and IPEC-J2 from pig jejunum [2]. The majority of studies have been carried out on IPEC-J2, which generated in 1989 by Berschneider [23] and is considered a useful model for ion transport research. However, except for an abstract form the annual meeting of the American Gastroenterological Association, few studies have documented the generation of a stable, non-transformed porcine intestinal epithelial cell line.

Immortalized cell lines have numerous advantages over primary cultures, particularly the retention of reasonably constant characteristics for following numerous passages [24]. Human telomerase reverse transcriptase (hTERT) is the catalytic subunit of the telomerase enzyme, which together with the telomerase RNA component (TERC), comprise the telomerase ribonucleoprotein complex. Telomerase activation is a critical step in cellular immortalization and tumorigenesis [25], [26], and hTERT alone has been found to be necessary and sufficient for inducing the telomerase activity [27]. Overexpression of hTERT has been previously used as a strategy for immortalization of human retinal pigment epithelial cells [27], swine vascular endothelial cells [28] and the cattle type II alveolar epithelial cell line [29].

In this study, the hTERT gene was successfully introduced into swine small intestinal epithelial cells, resulting in stable hTERT expression. After screening and identification, an immortalized cell line designated ZYM-SIEC02 was established. Immortalized ZYM-SIEC02 cells retained morphological and functional characteristic typical of primary small intestinal epithelial cells, and can be used as an *in vitro* model for mechanistic studies of pathogenic infections.

## Materials and Methods

### Ethics Statement

All animal experiments were approved by Care and Use of Animals Center, Northwest A & F University. This study was carried out in strict accordance with the Guidelines for the Care and Use of Animals of Northwest A & F University. Every effort was made to minimize animal pain, suffering and distress and to reduce the number of animal used.

### Reagents, antibodies and experimental animals

DMEM/F12 and FBS were purchased from Gibco. EGF, ITS-G and Lipofectamine Plus were products of Invitrogen. The WST-1 Cell Proliferation and Cytotoxicity Assay Kit was obtained from Beyotime, Shanghai, China. The Annexin V-FITC Apoptosis Detection Kit was a product of Calbiochem, Darmstadt, Germany. The TRAP-silver staining Telomerase Detection Kit was purchased from KeyGEN Biotech, Nanjing, China. The following primary antibodies were used: Pan-cytokeratin (clone AE1/AE3, AbD Serotec, Oxford, UK), mouse anti-Cytokeratin 18 (clone CY90, Sigma, St. Louis, MO, USA), rabbit anti-Sucrase-isomaltase (clone H-123, Santa Cruz, Heidelberg, Germany), rabbit anti-E-cadherin antibody (Genscript), mouse anti-OCLN (Clones 1G, AbD, Oxford, UK), rabbit anti-villin and anti-ZO-1 (Bioss, Beijing, China).

Healthy unsuckled 1-day-old Landrace piglets were purchased from the pig farm of Northwest Agriculture & Forestry University.

Nude mice at 4 weeks old of age were purchased from the Experimental Animal Center of The Fourth Military Medical University (Xi'an, China) and maintained in pathogen-free conditions.

### Isolation and culture of primary intestinal epithelial cells

Small intestines were collected from two 1-day-old, unsukled piglets. The mid-jejunum was dissected from each piglet, cut into 8–10 cm segments, then placed into ice-cold phosphate buffered saline (PBS) containing 200 U/ml of penicillin and 200 g/ml of streptomycin. The intestinal lumen was flushed with PBS using a 20 ml syringe until the liquid ran clear. The lumen was cut into small pieces with eye scissors, washed 3 times with PBS and once with DMEM/F12, and, cell pellets from each intestinal segment were collected by centrifugation at 800 *g* for 7 min. Cell pellets were resuspended in DMEM/F-12, washed twice with DMEM/F12, and resuspended in DMEM/F-12 containing 10 mM HEPES, 2 mM L-glutamine, 100 U/ml penicillin, 100 µg/ml streptomycin, 10 ng/ml EGF, ITS (insulin 1.0 g/l, transferrin 0.55 g/l and selenium 0.67 mg/l), and 5% heat-inactivated FBS and cultured at 37°C in a humidified incubator with 5% CO_2_. Cells reached confluence after 10–12 days and were then used for further experiments. The method of trypsin digestion with Citric acid was used to purify primary intestinal epithelial cells, purified epithelial cells were diluted at a density of 50 cells/ml and then, 100 µl suspensions were cultured to 96 well plates to select epithelial clones.

### Transfection and screening

After 2 passages, primary swine intestinal epithelial cells (pSIECs) were seeded onto 24-well plates at a density of 1×10^4^ cells/cm^2^. After 24 h, cells at 60–70% confluence were transfected with pCI-neo-hTERT by lipofection. Forty-eight hours following transfection, a selecting dose of G418 (600 µg/ml) was added to the culture medium, and the G418-containing. Medium was replenished every another day. After 12 days, drug-resistant cells were selected and maintained in 300 µg/ml of G418 to ensure a stable selected population, and positive clones were enlarged in further culture.

### Scanning and Transmission electron microscopy

Samples for scanning electron microscopy (SEM) and transmission electron microscopy (TEM) were prepared as described previously [30]. Briefly, cells were collected and fixed with 2.5% glutaraldehyde in 0.1 M sodium cacodylate buffer, pH 7.4 for 1 h, then post-fixed with 0.2% uranyl acetate. After dehydration in a graded series of ethanol, samples were gold-coated for SEM or embedded in EpON 812 resin for TEM. SEM specimens were visualized using a S-3400N scanning electron microscope at a 5 kV potential and TEM specimens were visualized on a JEM-1400 transmission electron microscope.

### Immunofluorescent staining

Immunostaining for keratin was performed according to the method of Shierack et al [2]. Briefly, cells were fixed in acetone for 10 min at −20°C and blocked with 1% bovine serum albumin (BSA). After washing 3 times in PBS, samples were immunolabeled using antibodies against pan-cytokeratin (1∶400) or cytokeratin 18 (1∶400) overnight at 4°C. Subsequently, samples were incubated with secondary antibody (FITC-conjugated goat anti-mouse IgG (H+L)) at room temperature (RT) for 1 h. The samples were mounted and then imaged using fluorescent microscopy.

### Western Blot analysis

Confluent cells were rinsed twice with PBS, harvested by scraping into RIPA buffer containing protease inhibitors, and sonicated for 10 s. Equibalent amounts of proteins were separated by 10% SDS-PAGE, and transferred to a PVDF membrane (Millipore, Massachusetts, USA). Membranes were washed 4 times for 5 min each with PBS, blocked with 5% non-fat milk (Yili Industrial Group Co., Ltd, Hollyhock, China) in PBST (PBS containing 0.5% Tween 20) for 1 h, at RT. After washing, the membranes were incubated with an anti-GAPDH antibody or primary antibodies diluted in the blocking solution according the manufacturer’s instructions for 1 h at 37°C with shaking (or overnight at 4°C). Secondary antibody (HRP-labeled goat anti-mouse or anti-rabbit IgG (H+L) ) (Bioss, Beijing, China) diluted at 1∶5,000 in blocking solution was added to the membranes and was incubated for 1 h, at RT. Antigen-antibody complexes were detected using the Western Light kit (Advansta, Menlo Park, USA).

### Flow cytometry

Cells were collected, fixed with 70% ethanol, and stored at 4°C until analysis. After removing the supernatants and washing, cells were resuspended in PBS at a concentration of 1×10^6^ cells/ml, then 100 µl of cell suspensions was placed into preparatory tubes combined with 200 µl DNA-PREPTMLPR, and incubated for 30 s. Samples were then mixed with 2 ml of DNA-PREPTMLPR staining reagent (propidium iodide solution) and incubated at RT for 30 min. Cell cycle phases were examined by flow cytometry, and data were processed using SYSTEM II software.

Cell apoptosis was detected using an Annexin V-FITC Apoptosis Detection Kit (Calbiochem, Darmstadt, Germany). Cells (5×10^5^) were collected in PBS and centrifuged twice at 100 *g* for 5 min. The supernatants were discarded and 0.5 ml 1× binding buffer and 1.25 µl Annexin V-FITC were added to the cells, followed by incubation at RT for 15 min, protected from light. Cells were washed twice with 1× binding buffer, then resuspended in binding buffer to which 10 µl of propidium iodide was added; cells were then incubated for 10 min. The percentage of apoptotic cells was measured by flow cytometry, and the data were processed using WinMDI2.9 software.

### Telomerase activity analysis

pSIECs (passage 3), ZYM-SIEC02 cells (passage 50) and A549 cells (a lung adenocarcinoma cell line) were trypsinized, washed twice with PBS, and pelleted by centrifugation at 2,000 rpm for 5 min. Telomerase activity was analyzed using a TRAP-silver staining Telomerase Detection Kit (KeyGEN Biotech, Nanjing, China) according to the manufacturer’s instructions.

### Soft agar assay

Growth in soft agar is the minimum requirement to demonstrate in *vitro* transformation. A lower layer of 0.5% agar gel was prepared in 24-well plates at 4°C. Subsequently, ZYM-SIEC02 cells were trypsinized and resuspended at concentrations of 5×10^3^, 1×10^4^ and 2×10^4^ cells/ml in DMEM/F-12 containing 20% FBS. Thereafter, 2 ml of 0.5% agar was added to 1 ml of cell suspension (final concentration: 0.33% agar) and cell suspensions (1 ml) were overlaid onto the solidified lower layer and incubated at 37°C in a humidified atmosphere with 5% CO_2_ for 2 weeks. Colonies were then analyzed under a microscope. We considered the presence of even a single colony an indication that cells were capable of anchorage-independent growth.

### Karyotype analysis

The number of chromosomes of transfected cells were determined by karyotype analysis performed as previously described by Hong et al [28].

### Tumorigenicity assay

To evaluate *in vivo* tumorigenic potential, primary cells and ZYM-SIEC02 cells were trypsinized and resuspended in DMEM/F-12 at a concentration of 1×10^6^ cells/ml, and 0.2 ml of the cell suspension was injected subcutaneously into the flanks of 6 nude mice (4 weeks old). As a positive control, equivalent number of EMF6 cells (a breast cancer cell lines derived from BALB/c mice) were injected into the flanks of 6 nude mice, both group of mice were monitored for 1 month to observe tumor formation.

### Paraffin sections

After sacrificing the mice, tissue samples were removed from both control animals and from injected nude mice, then fixed in neutral buffered 10% formalin and processed into paraffin-embedded blocks for haematoxylin eosin staining. The study of histological structures was performed using light microscopy.

### Proliferation assay

The growth curves were determined as previously described [28]. ZYM-SIEC (passage 50) and pSIECs (passage 3) were seeded in 24-well plates at 1×10^4^ cells/well. Cells were digested with trypsin and counted each day for 8 days. Three independent experiments were performed in triplicates. The growth curves of the cells were plotted.

The proliferation of ZYM-SIEC02 cells in response to medium supplements was evaluated as previously described [2]. Briefly, ZYM-SIEC-02 cells were seeded into 96-well plates at 5×10^3^ cells per well and cultured for 24 h. Cell culture medium and non-adherent cells were removed and replaced with fresh medium containing either FBS (2%, 5%, 10%, 15%, 20%), EGF (5 ng/ml), or ITS for an additional 24 h. Cell growth determined using the WST-1 Cell Proliferation and Cytotoxicity Assay Kit (Beyotime, shanghai, China) during the last 24 h of incubation. Cell proliferation was quantified using a Multiskan FC Microplate Photometer (Thermo Scientific, USA). Three independent experiments were performed in triplicates.

### Bacterial invasion

Invasion assays were performed essentially as previously described [31]. *Salmonella enterica serovars Typhimurium*(*S. Typhimurium*) was grown in L-broth to an optical density at 600 nm (OD_600_) of approximately 1. After centrifugation and washing, bacteria were resuspended in cell culture medium and diluted to a multiplicity of infection (MOI) of 10∶1 (*Salmonella*: host cells) in a 12-well plate. Confluent monolayers were infected at 37°C, 5% CO_2_ for 1 h to allow bacterial entry. After removal of the extracellular bacteria, cultures were incubated for an additional hour in medium containing 50 µg/ml gentamincin to kill any remaining extracellular bacteria. Infected cells were washed twice with PBS, lysed with 0.1% Triton ×−100 in deionized water. Dilutions of cell lysates were plated onto L-broth agar plates for quantification of intracellular bacteria. Infections were carried out in triplicate.

### Exposure of ZYM-SIEC02 cells to inflammatory stimuli

Treatments included control (uninfected cells), LPS (L-2880, from *E.coli* 055:B5; Sigma Chemical Co., St. Louis, MO; 1 µg/ml), or *Salmonella*. Bacteria were grown as described for invasion assays. After the bacterial population was estimated by spectrophotometry at OD600, bacteria were pelleted and resuspended in DMEM/F-12 growth media lacking FBS and antibiotics. Confluent ZYM-SIEC02 cells were washed twice with PBS and 1 ml of media alone (control), bacteria was added to the wells and plates were further incubated for 1 h. Cell culture medium from control treatments or cultures exposed to bacteria was replaced with fresh media containing 50 µg/ml gentamicin. Medium was removed from wells 4 h after LPS or bacteria exposure for determination of IL-8 and TNF-α secretion, and adherent cells were washed 3 times with PBS and lysed with Trizol reagent (Invitrogen, Carlsbad, USA).

### Inhibition of LPS-mediated response

*E.coli* LPS 055:B5 (0.0125, 0.25 or 5 µg/mL) were pre-incubated with or without 10 µg/mL of polymyxin B sulfate (Sigma, Shanghai, China) for 3 hours. ZYM-SIEC02 cells were then washed 3 times with PBS and lysed with Trizol reagent (Invitrogen, Carlsbad, USA).

### Real time PCR

Total RNA was isolated according to the manufacturer’s instructions. The quality and purity of total RNA was determined by optical density (OD) at 260 nm and 280 nm wavelengths using a spectrophotometer. Total cDNA was synthesized using M-MLV Reverse Transcriptase (Takara, Dalian, China) according to the manufacturer’s instructions. Relative gene expression was determined by qPCR using an iQ5 Thermo Cycler (Bio-Rad). 20 µl real-time PCR reactions were carried out using 2 µl of diluted cDNA as template and iQTM SYBR Green Supermix (Takara, Dalian, China) according to the manufacturer’ instructions. Thermal cycling parameters were utilized according to manufacturer recommendations by 40 cycles of amplification with alternating 5 s 95°C denaturation and 30 s 56°C anneal/extension. Specific genes were amplified with DNA polymerase using the primers listed in Table 1.

**Table 1:** List of primers used for Real-time PCR.

Genes	Primer sequences (5′–3′)	Reference/accession
TLR-4	F.GCCATCGCTGCTAACATCATC	1
	R. CTCATACTCAAAGATACACCATCGG	
IL-8	F. CTGGCTGTTGCCTTCTTG	NM_213867.1
	R. TCGTGGAATGCGTATTTATG	
TLR-6	F. AACCTACTGTCATAAGCCTTCATTC	1
	R.GTCTACCACAAATTCACTTTCTTCAG	
TNF-α	F. CGCATCGCCGTCTCCTACCA	NM_214022
	R. CTGCCCAGATTCAGCAAAGTCCA	
IL-6	F. TGGATAAGCTGCAGT CACAG	2
	R. ATTATCCGAATGGCCCTCAG	
β-actin	F. CAAGGACCTCTACGCCAACAC	DQ845171.1
	R. TGGAGGCGCGATGATCTT	

1 [49]; 2 [56].

### ELISA assay

Infection of cell monolayers with Salmonella typhimurium was performed as described for invasion assays. Supernatants from infected and uninfected cells were harvested after 4 h. *ELISA* were performed according to the manufacturer’s protocols (Swine IL-8 or TNF- *ELISA* Kit, Invitrogen, Camarillo, CA, USA).

### Statistical analysis

Relative gene expression of chemokines and cytokines in ZYM-SIEC02 cells was determined from real time PCR data using the ΔΔCT method as previously described [32], [33] Data were expressed as mean ± (SEM). Differences between groups were examined for statistical significance using the Student’s t-test. A *P* value <0.05 was considered as statistically significant.

## Results

### Immortalized intestinal epithelial cells maintained the morphological features of the primary cells

**Figure 1:**
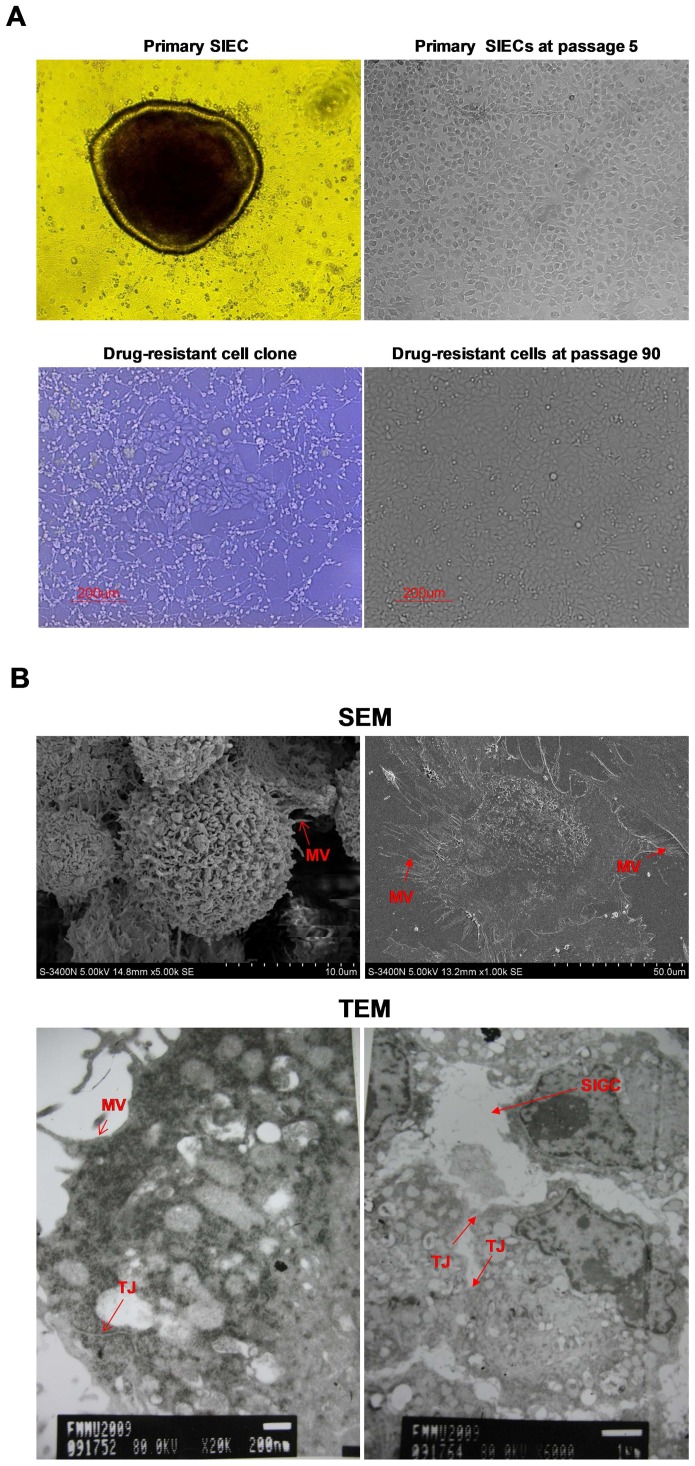
Morphological features of pSIECs and ZYM-SIEC02 cells. **A**. Cellular morphology of pSIECs at 7 days in culture (100×) and at passage 5 (100×); a drug-resistant cell clone (100×) and ZYM-SIEC02 cells at passage 90 (100×).No obvious morphological differences were observed between ZYM-SIEC02 cells and primary SIECs. **B**. Electron micrographs of monolayer cultures of ZYM-SIEC02 cells indicate microvilli (MV) in 3 dimensions andin monolayer culture; tight junctions (TJ) and a small intestine glandular configuration (SIGC) (Red arrow).

Cell material obtained from porcine mid-jejunum consisted of cell aggregates or organoids that adhered to collagen-coated culture flasks and formed circular proliferating foci (Fig. 1A, upper left). Initial expansion of the foci resulted in fusion of the cell plaques, forming a confluent cell layer within 10–12 days due to the presence of residual multicellular organoids dispersed in the newly formed monolayer. During the first passage, epithelial cell sheets exhibited a homogeneous cobblestone-like morphology. No obvious morphological changes were observed during prolonged passage in culture, defined as 8–10 passages at a split ratio of 1∶2 over 4–5 weeks (Fig. 1A, upright). Cells reached confluence within 2–3 days during the early passages (p1–p6). However, a progressive slowdown in the proliferation rate was observed until cells reached senescence. Eleven passages later, cells became enlarged and the cobblestone-like morphology was lost, even though the expression of cytokeratin 18 was maintained (data not shown).

In order to establish an immortalized porcine intestinal epithelial cell line, pSIECs at passage 2 were transfected with the plasmid of pCI-hTERT-neo, and selected with G418 for 2 weeks, at which time we obtained a drug-resistant clone, which we designated ZYM-SIEC02 (Fig. 1A, lower left). ZYM-SIEC02 cell exhibited similar morphological features as compared to control pSIEC, even at passage 90 pSIECs (Fig. 1A lower right). To date, the established ZYM-SIEC02 cell line has been cultured for more than 100 passages at split ratios of 1∶2 or 1∶3.

Ultrastructural analyses indicated that ZYM-SIEC02 cells in culture maintained the structures of microvilli and apical tight junctions (Fig. 1B). Interestingly, a small intestine glandular configuration (Fig. 1B, lower right) was also observed. In addition, the majority of cells possessed sponge-like nucleoli with a high nucleus: cytoplasm ratio, suggests that the cells have a robust proliferative activity.

### Epithelial cell-specific markers are stably expressed in immortalized ZYM-SIEC02 cells

**Figure 2:**
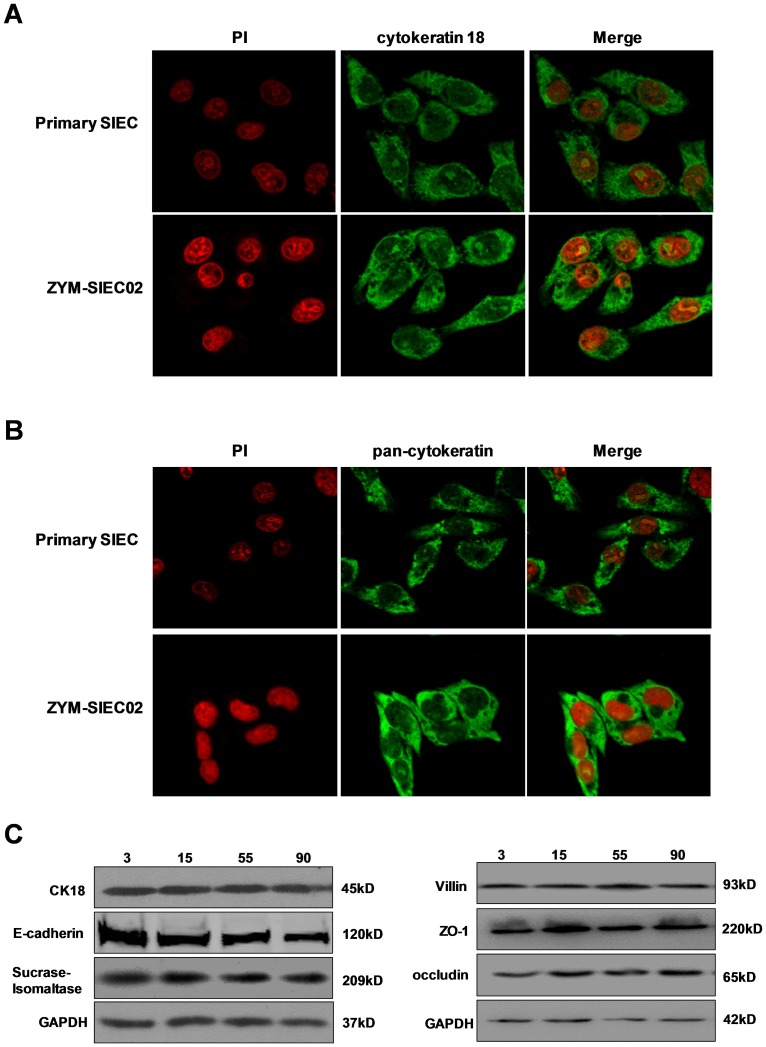
Immunostaining and western blot analysis of ZYM-SIEC02 cell cultures. **A**. pSIECs and ZYM-SIEC02 cells were stained with cytokeratin 18 antibodies (green) and propidium iodide (red). **B**. pSIECs and ZYM-SIEC02 cells were stained with pan-cytokeratin antibodies (green) and propidium iodide (red). **C**. expression of cytokeratin 18, E-cadherin, SI, villin, ZO-1 and occludin were determined by western blot analysis. Cytokeratin 18, pan-cytokeratin, E-cadherin, SI, villin, ZO-1 and occludin were expressed in both pSIECs (at passage 3) and ZYM-SIEC02 cells (at passages 15, 55, and 90).

ZYM-SIEC02 cells were characterized using immunofluorescence and western blot analysis. Immunostaining for cytokeratin indicated characteristic disposition of intermediate filaments, and intracellular expression of cytokeratin 18 (Fig. 2A) and pan-cytokeratin (Fig. 2B).

Western blot analysis confirmed the expression of epithelial cell markers in ZYM-SIEC02 cells. A cytokeratin 18 antibody recognized a 45 kD protein in homogenates from primary porcine enterocytes, as well as in those from early (passage 5), intermediate (passage 55) and late passages (passage 90) of ZYM-SIEC02 cells. ZYM-SIEC02 cells also expressed the epithelial cell marker E-cadherin (∼120 kD), which plays an important role in the maintenance of tight junctions. Moreover, the expression of tight junction proteins ZO-1 and occludin confirmed that ZYM-SIEC02 cells formed intercellular tight junctions. In order to determine whether these cells were differentiated, sucrase-isomaltase (SI, 209 kD), a marker of differentiation expressed in the intestinal brush border, was detected as described previously [34] (Fig. 2C). In addition, both pSIECs and ZYM-SIEC02 cells expressed detectable villin protein (93 kD), confirming the ultrastructural analysis (Fig. 1B).

### Enhanced proliferation and decreased apoptosis in immortalized cells

**Figure 3:**
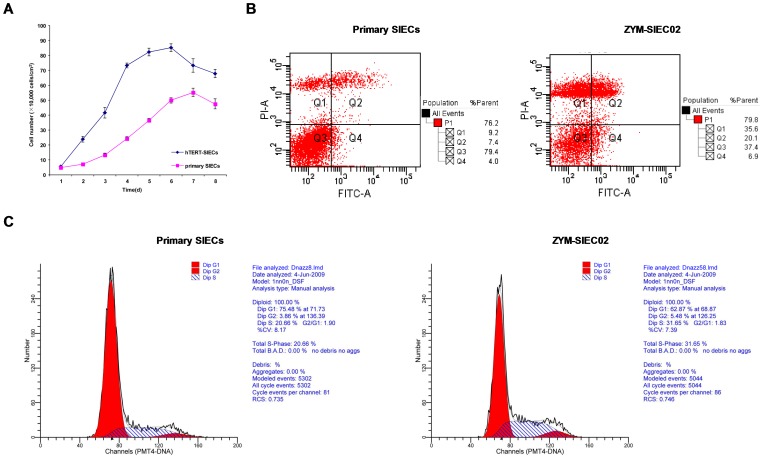
Growth curves, apoptosis and cell cycle analysis of SIECs before and after hTERT transfection. **A**. Growth of pSIECs and ZYM-SIEC02 cells. Data are represented as the mean ± SD of 3 independent experiments. **B**. Apoptosis analysis of control pSIECs and ZYM-SIEC02 cells, The percentage of apoptosis in pSIECs and ZYM-SIEC02 cells under basal growth conditions was 26.9% and 11.4% (Q2+Q4), respectively. **C**. Cell cycle distributions of control SIECs and ZYM-SIEC02 cells, the percentages of pSIECs and ZYM-SIEC02 cells in S phase were 20.66% and 31.65%, respectively.

The growth curves of ZYM-SIEC02 cells and control pSIEC are shown in Figure 3A. In order to ascertain whether hTERT expression affected cell proliferation or cell cycle progression, cell cycle distribution and basal levels of apoptosis in pSIECs and ZYM-SIEC02 cells were determined by flow cytometry. The percentages of apoptotic cells in pSIECs and ZYM-SIEC02 cultures were 26.9% and 11.4% (Q2+Q4), respectively (Fig. 3B). The percentages of pSIECs and ZYM-SIEC02 cells in S phase were 20.7% and 31.7%, respectively (Fig. 3C), indicating that the immortalized ZYM-SIEC02 cell line exhibits increased proliferative activity and a lower incidence of apoptosis compared to that of primary swine intestinal epithelial cells.

### Increased telomerase activity in transfected cells

**Figure 4:**
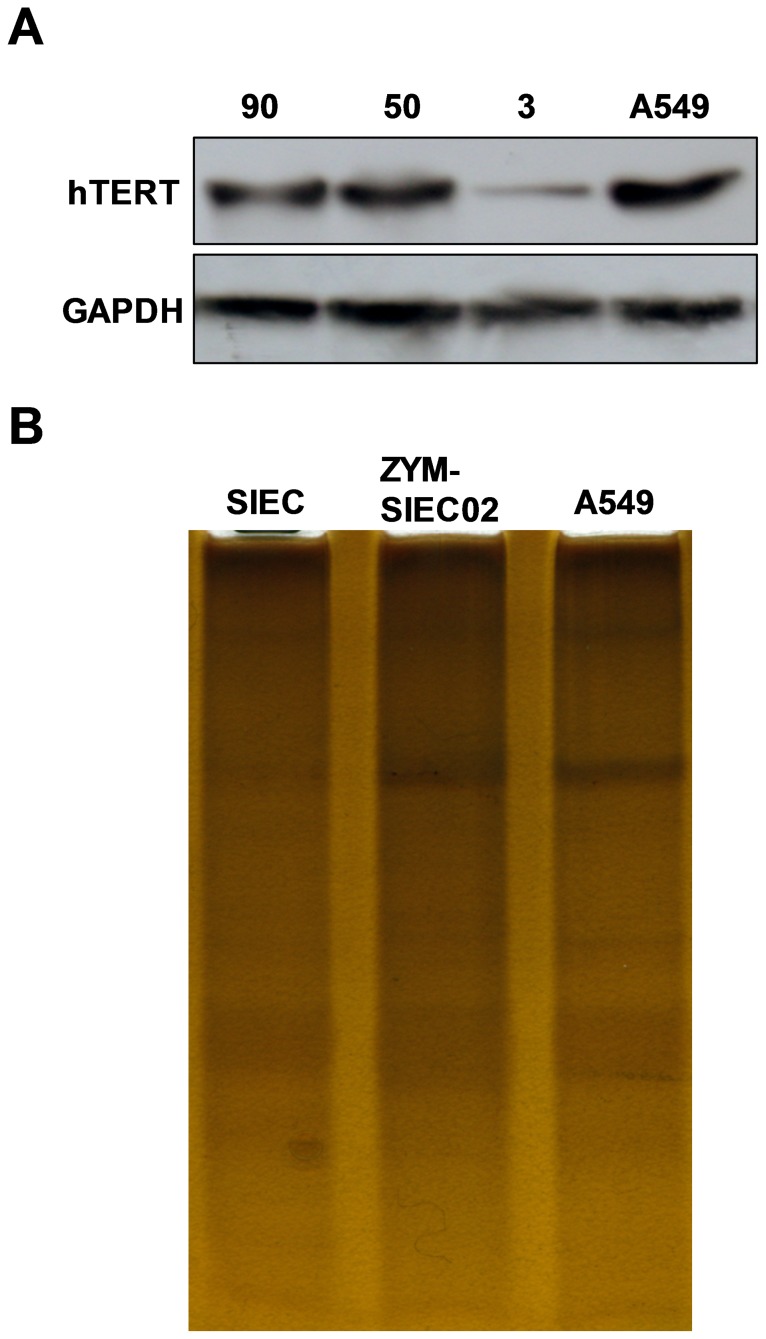
Detection of Telomerase activity. **A.** Expression of hTERT protein was detected by western blot analysis. Expression of hTERT was readily detected in ZYM-SIEC02 cells at passage 50 and 90, but was barely detectable in pSIECs at passage 3. The A549 cell line was used as a positive control. **B**. Silver staining method was used to detect telomerase activity. The laddering patterns indicated that ZYM-SIEC02 cells display higher telomerase activity as compared to pSIECs and A549 cell line.

Telomerase activity in both pSIECs and ZYM-SIEC02 cells was detected by Western blot and a TRAP-silver staining Telomerase Detection Kit. There were no significant differences observed in the level of hTERT protein in ZYM-SIEC02 cells at early or late passage, but ZYM-SIEC02 cells exhibited higher hTERT expression and activity than pSIECs (Fig. 4A, B).

### The newly established ZYM-SIEC02 cell line is immortalized but not transformed

**Figure 5:**
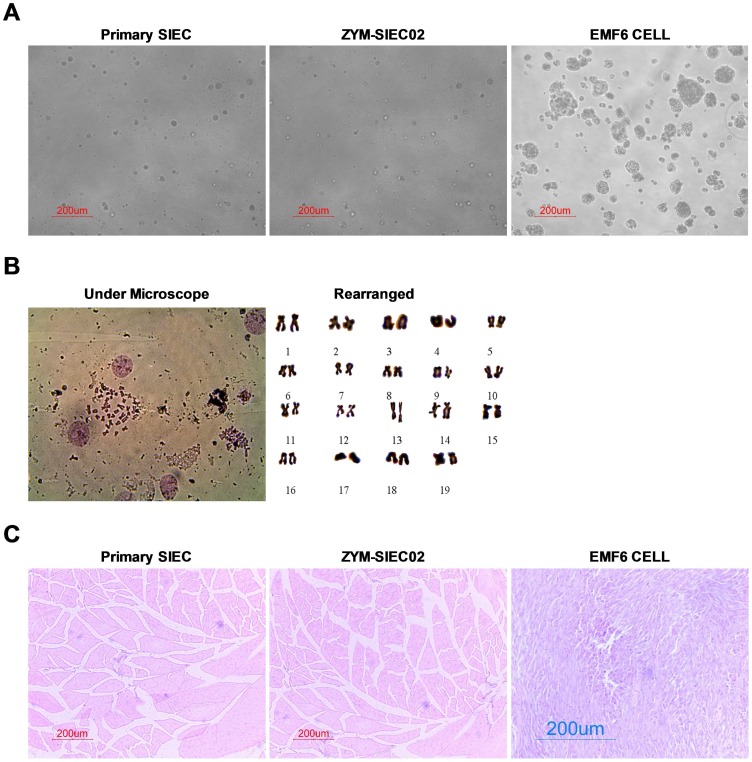
Tumorigenicity assay of ZYM-SIEC02 cells. **A.** Representative images of pSIECs and ZYM-SIEC02 cells growing in soft agar. Neither cell line formed colonies after 2 weeks in culture, indicating that the cells did not exhibit anchorage-independent growth (100×). **B**. Karyotype analysis of ZYM-SIEC02 cells at passage 55, indicated that the cells contained 38 chromosomes, consistent with a diploid karyotype. **C**. Tumor formation assay. pSIECs and ZYM-SIEC02 cells were injected subcutaneously into nude mice. After one month, neither group of mice had developed tumors, in contrast, mice injected with EMF6 cells rapidly developed tumors within 8 days. Histological examination indicated morphologically normal tissue below the injection site of pSIECs (passage 3) and ZYM-SIEC02 cells (passage 55), but a dense cellular mass below the EMF6 cell injection site.

Anchorage-independent growth was tested using a clonogenic soft agar assay. While EMF6 cells formed numerous colonies, ZYM-SIEC02 cells and pSIECs did not form colonies after 2 weeks in soft agar (Fig. 5A), indicating that ZYM-SIEC02 cells did not exhibit anchorage-independent growth.

Karyotype analysis of ZYM-SIEC02 cells at passage 60 was performed to determine whether transduction with hTERT affected the chromosomal status. We observed a normal complement of chromosomes in ZYM-SIEC02s with a modal chromosome number of 38 (Fig. 5B).

A definitive functional assay for determining tumorigenicity was carried out by injecting either ZYM-SIEC02 cells or tumorigenic EMF6 cells subcutaneously into nude mice. Tumors were observed with 7–8 days in all mice injected with EMF 6 cells, while no tumors developed in mice injected with ZYM-SIEC02 cells even after 1 month. Further histological examination revealed a normal tissue structure at the site of ZYM-SIEC02 cells or pSIEC cell injection, whereas dense cellular masses were observed at the EMF6-injection site (Fig. 5C).

### ITS is beneficial to cell growth

**Figure 6:**
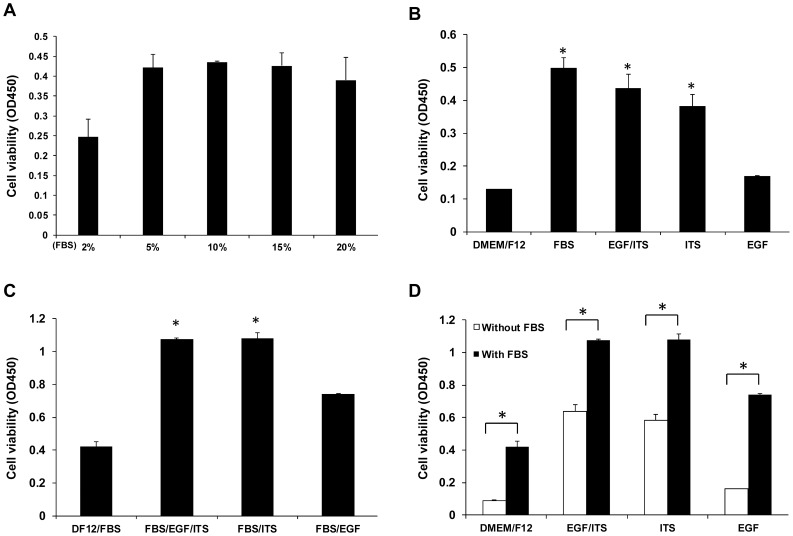
Optimization of culture medium for ZYM-SIEC02 cells. **A**. ZYM-SIEC02 cells were cultured for 24 h with 2%, 5%, 10%, 15%, or 20% FBS. 5% FBS was found to be the optimal concentration. **B**. Addition of 5% FBS or ITS, and/or EGF to the cell medium individually, compared with DMEM/F12 alone; Addition of ITS or ITS/EGF enhanced cell growthsimilar to the addition of 5% FBS, while addition of EGF alone did not have a significant effect. **C**. ITS/EGF or ITS significantly enhanced cell growth with the use of cell culture medium containing 5% FBS. **D**. Addition of ITS, EGF, or EGF/ITS to DMEM/F12 had little effect on the proliferation rate compared to the addition to 5% FBS. * *P*□0.05 versus basal conditions.

To optimize the culture conditions of our newly immortalized epithelial cells, medium supplements for growth of the cell line were tested. Based on cell growth responses, a concentration of 5% fetal bovine serum (FBS) was found to be optimal (Fig. 6A). To further confirm whether medium additives promote cell proliferation, 5% FBS or ITS (1.0 g/l insulin, 0.55 g/l transferrin, and 0.67 mg/l selenium) and/or epidermal growth factor (EGF, 10 ng/ml) were added to the culture medium. We found that, ITS alone or a combination of ITS and EGF enhanced cell growth equivalent to that induced by 5% FBS alone (Fig. 6B). Moreover, the addition of ITS to the culture medium containing 5% FBS further promoted cell proliferation while EGF alone had no effect (Fig. 6C, D).

### Invasion efficiency of Salmonella typhimurium in the ZYM-SIEC02 cell line

**Figure 7:**
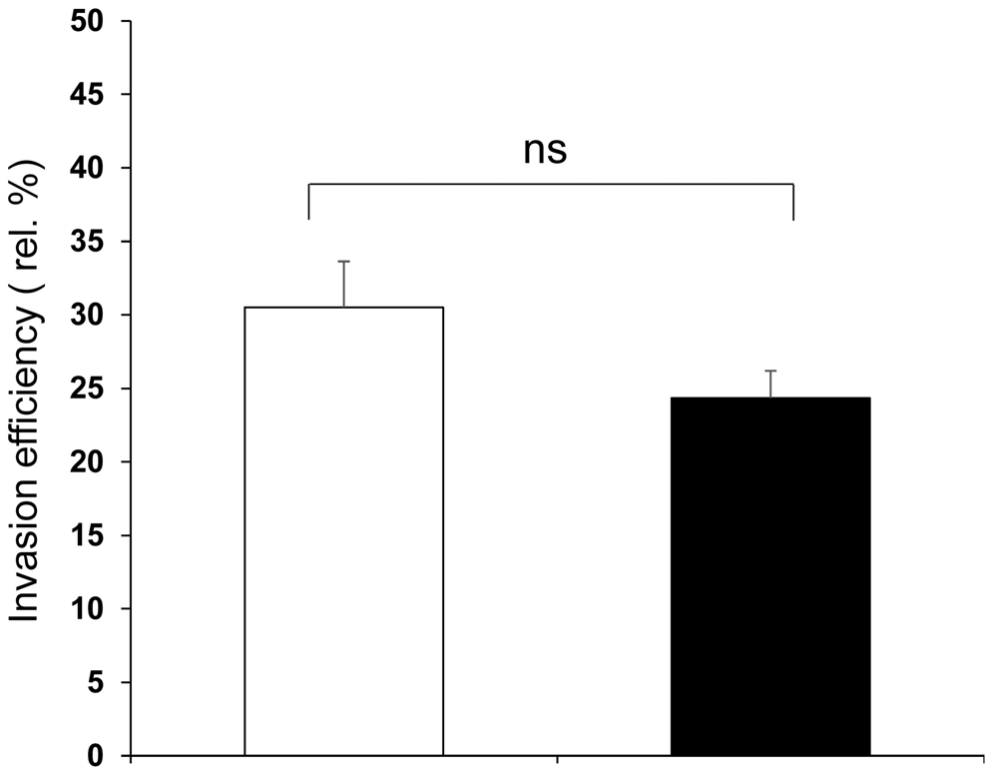
Invasion efficiency of *Samonlla Thpyimurium* in the ZYM-SIEC02 cell line. Confluent monolayers of ZYM-SIEC02 cells and pSIECs were infected with a MOI of 10∶1 (*S. Typhimurium* to host cells) for 1 h, and incubated in media containing gentamicin for an additional hour. Cells used were pSIECs (open bars) and ZYM-SIEC02 cells (filled bars). Data shown are normalized to the efficiency (relative percent of input bacteria) and representative of 5 independent experiments performed in triplicate.

*Salmonellae* are facultative intracellular pathogens. An essential step in Salmonella pathogenicity is the formation of an intracellular *Samonella*-containing vacuole in which the pathogen replicates [2]. To determine whether ZYM-SIEC02 cells are susceptible to *Samonella typhimurium* invasion in *vitro*, intracellular bacteria were quantified in order to calculate invasion efficiency. As shown in Fig. 7, both pSIECs and ZYM-SIEC02 cells could engulf *Salmonella* during the invasion process.

### ZYM-SIE02 cells maintained the immune functions of pSIECs

**Figure 8:**
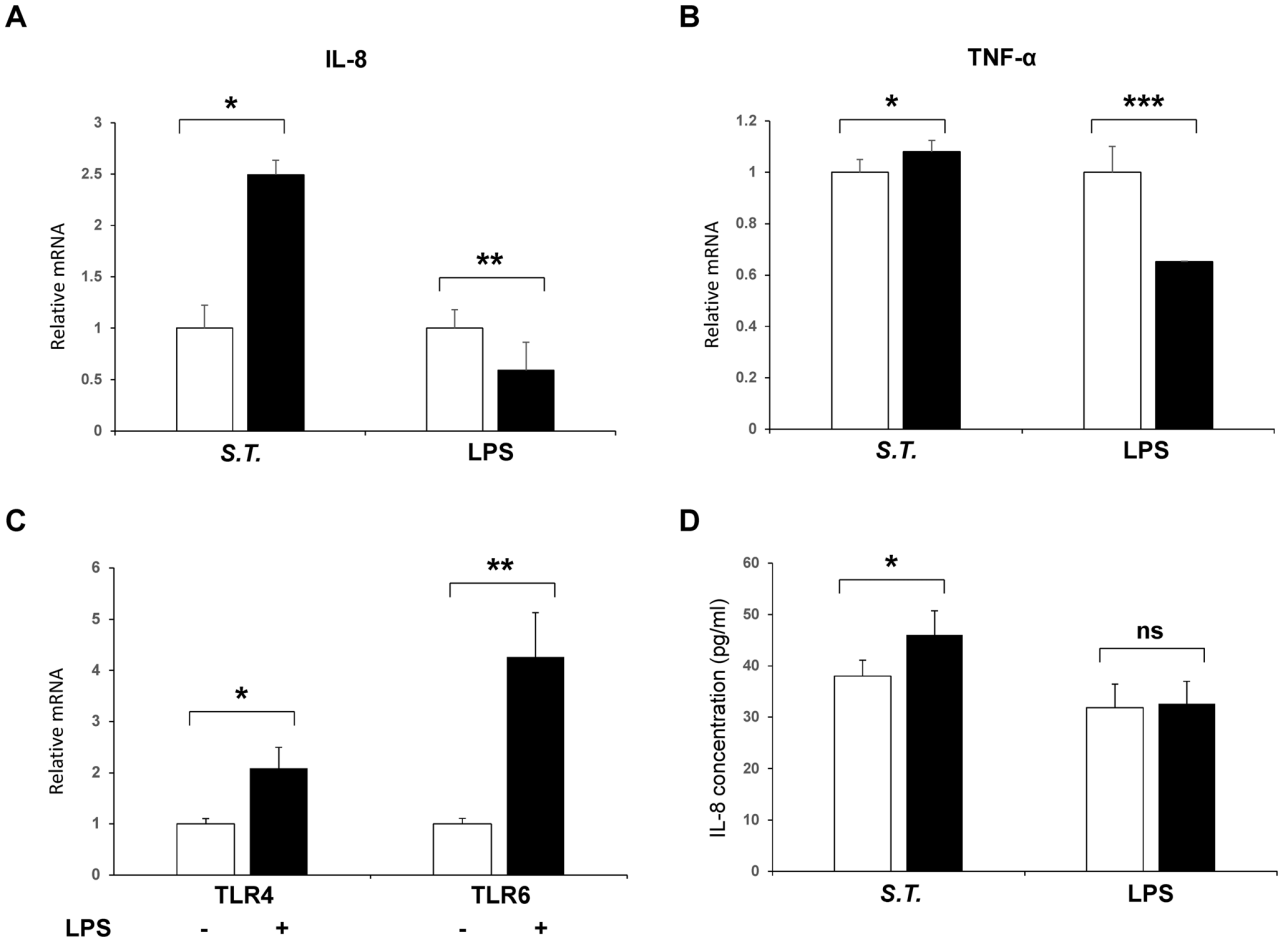
Cytokine expression in ZYM-SIEC02 cells infected with *Salmonella typhimurium* and LPS. Confluent ZYM-SIEC02 cell monolayers were infected for 4 h with *S. Typhimurium* or LPS. **A**. Relative mRNA expression of IL-8 in ZYM-SIEC02 cells were upregulated approximately 2.5-fold within 4 h of *S. Typhimurium* challenge relative to control, while the cells that stimulated with LPS demonstrated a significant reduction; **B**. TNF-α mRNA expression was a modest increased by *S. Typhimurium* stimulation, while the cells that stimulated with LPS demonstrated a significant reduction; **C**. TLR4 and TLR6 mRNA expression were upregulated following stimulation with LPS. D. IL-8 protein expression in ZYM-SIEC02 cells infected with *Salmonella typhimurium* and LPS**.** ZYM-SIEC02 cells were incubated with *S. Typhimurium* at a MOI of 100∶1 followed by an incubation in media containing gentamicin, and then cell culture supernatants were harvested after 4 h. ZYM-SIEC02 cells were stimulated with LPS, and cells were harvested after 4 h. IL-8 concentrations were determined by ELISA. The data shown are means±SEM of 3 independent experiments. * *P*<0.05;** *P*<0.01; *** *P*<0.001; ns, not significant.

To further verify the immunomodulatory responses of the ZYM-SIEC02 cell line, we evaluated the expression of the inflammatory factors and chemokines expressed in intestinal epithelial cells that are known to be involved in the immune response to pathogenic infection. ZYM-SIEC02 cells were used to evaluate the effect of *S. Typhimurium* and LPS on IL8 and TNF-α mRNA expression. As shown in Fig. 8, the expression of IL-8, TNF-α was upregulated following *S. Typhimurium* challenge and IL8 expression was upregulated approximately 2.5-fold within 4 h of *S. Typhimurium* challenge relative to control, while TNF-α mRNA expression was a modest increased by *S. Typhimurium* stimulation. Interestingly, cells that stimulated with LPS demonstrated a significant reduction in IL-8 and TNF-α mRNA expression compared to controls (Fig. 8A, B). To determine if this functional immune response were via TLR4 recognition on intestinal epithelial cells, we choose to detect TLR4 and TLR6 mRNA expression following stimulation with LPS. The result showed that TLR4 and TLR6 were upregulated (Fig. 8C).

In order to determine if challenged ZYM-SIEC02 cells exhibit differential protein expression during an inflammatory response, IL-8 and TNF-α secretion were evaluated by ELISA. Exposure to *S. Typhimurium* resulted in an initial trend toward an increase in IL8 secretion (Fig. 8D). However, the lack of response of swine intestinal epithelial cell in* vitro* to stimulate with LPS was found in terms of IL8 production. Moreover, TNF-α secretion in ZYM-SIEC02 cells remained undetectable while challenged with both *S. Typhimurium* and LPS (data not shown).

**Figure 9:**
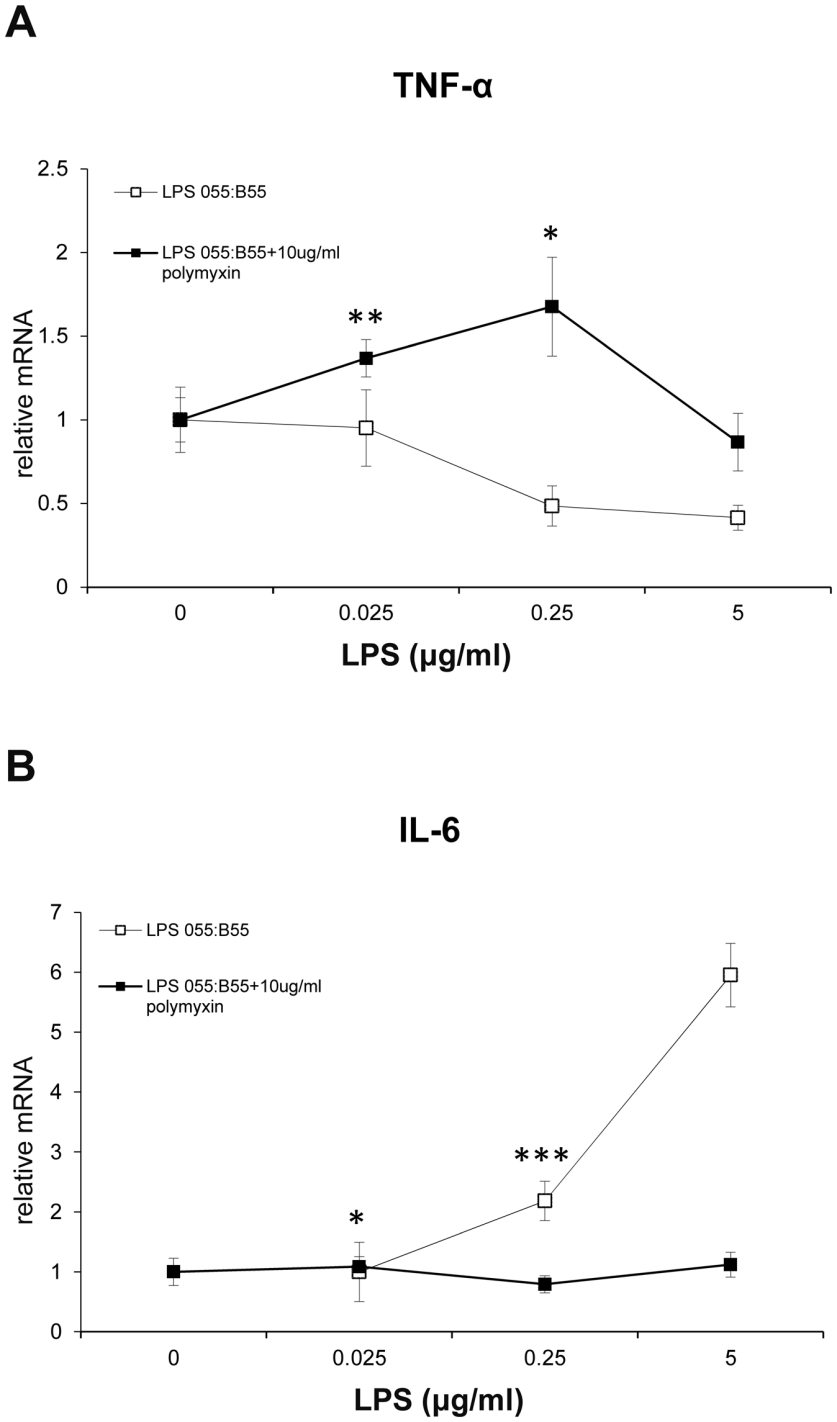
Inhibition of LPS-mediated response. ZYM-SIEC02 cells were stimulated with the indicated dose of *E.coli* 055:B55 LPS in the presence or absence of polymyxin B sulfate(Sigma) for 3 hours. A. Polymyxin B increased TNF-α mRNA expression when the concentration of LPS was lower than 250 ng/ml. B. Polymyxin B prevented the increase in IL-6 mRNA expression. The data shown are means±SEM of 3 independent experiments. * *P*<0.05;** *P*<0.01; *** *P*<0.001.

Interestingly, antagonism of TLR4 signaling with polymyxin B prevented the increase in IL-6 mRNA expression but increased TNF-α mRNA expression when the concentration of LPS was lower than 250 ng/ml (Fig. 9A). As expected, ZYM-SIEC02 cells responded to LPS in a dose-dependent manner and polymyxin B blocked LPS recognition by host cells inhibited this response (Fig. 9B).

## Discussion

In the present study, we describe the establishment of the ZYM-SIEC02 cell line and demonstrate that ZTM-SIEC02 cells exhibit normal epithelial cell morphological and functional characteristics similar to pSIECs. Morphological analysis showed a typical cobblestone-like morphology, apical tight junctions, and the presence of microvilli. In addition, functional analysis suggested that this cell line was not transformed and was able to respond to *S. Typhimurium* infection by upregulating expression and secretion of IL8. These results demonstrate that introducing the hTERT gene can be used to immortalize porcine intestinal epithelial cells, consistent with previous findings that stable overexpression of hTERT gene is sufficient to immortalize swine umbilical vascular endothelial cells [28], and cattle type alveolar epithelial cell line [29].

Cytokeratins play a pivotal role in cell differentiation and tissue specialization, functioning to maintain the overall structural integrity of epithelial cells. The expression of cytokeratin is considered as the marker of epithelial cells [2]. In this study, expression of cytokeratin 18 and pan-cytokeratin was detected in ZYM-SIEC02 cells, demonstrating that the cells were epithelial in origin. E-cadherin is a membrane-spanning protein found in epithelial cells and plays a crucial role in formation of cell-cell junction formations [35]. Here, we found that E-cadherin was expressed by both pSIECs and ZYM-SIEC02 cells, indicating that ZYM-SIEC-02 cells preserved this characteristic of epithelial cells. Moreover, the enterocyte marker sucrase-isomaltase (SI) was detected in both pSIECs and ZYM-SIEC02 cells. SI is a small-intestinal microvillus hydrolase that localizes to the apical membrane of adult intestinal enterocytes along the intestinal crypt-villus axis [36]. Deficiency in SI protein results in osmotic diarrhea due to an inability to hydrolyze intestinal disaccharides into component monosaccharides.

Therefore, we conclude that the newly established cell line is comprised of small intestine-derived epithelial cells, which in turn suggests that this cell line can be used in future research on disease inducing porcine diarrhea.

Typically, differentiated cells were taken for representing the intestinal villus tip cells, while the undifferentiated cells were used to mimic the basilar crypt cells of the intestines [37], [38]. Thus, the prototypical characteristics of differentiated porcine intestinal epithelial cells are the presence of tight junctions and distinct microvilli on their apical surfaces [38], [39]. Here, we demonstrated that porcine intestinal epithelial cells expressed markers typical of differentiated enterocytes, specifically, ZYM-SIEC02 cells were positive for E-cadherin, ZO-1, Occludin, villin, and sucrose isomaltase, which is the most reliable indicator of intestinal cell differentiation in vitro [40], indicating the presence of differentiated villus cells in the culture. IPEC-J2 cells were cultured for 1 days or 21 days, representing undifferentiated proliferating and highly differentiated IPEC-J2 cells, respectively [41], [42]. However, in our experiment, all the ZYM-SIEC02 cells we used were cultured to have a confluent monolayer within 3 or 6 days according to the density of cell cultures. In this regard, the characteristics of differentiated ZYM-SIEC02 cell were evidently due to the time that isolated and purified the primary porcine intestinal epithelial cells was long enough for the differentiation of pSIECs, which prepared for the lipofection. Thus, ZYM-SIEC02 cells remain the characteristic of differentiated pSIECs, and markers typical of differentiated enterocytes were detected. Moreover, we used tissue culture method to isolate epithelial cells from small intestine, it was unavoidable that the growth of fibroblasts over cultured primary intestinal epithelial cells, however, on the other side of the coin, the intestinal epithelial cell differentiation need the heterologous cell-cell contacts for *denovo* synthesis to trigger cell polarity and differentiation [40]. The addition of EGF may be another important inducement for differentiation as it plays a pivotal role in the regulation of intestinal epithelial proliferation and differentiation [43]. Over time in culture, cells maintained their epithelial morphology as well as expression of markers (8–10 passages for pSIECs and more than 90 passages in ZYM-SIEC02 cells). These characteristics were not affected by cryogenic freezing and retrieval (data not shown).

An early study by Hayflick et al demonstrated that normal human somatic cells have a finite replicative potential *in vitro* [44], and will stop dividing after a finite number of population doublings and enter senescence. Moreover, the lifespan of human cells *in vitro* is known to be dependent on telomere length and telomerase activity. Telomere shortening in humans is a prognostic marker of disease risk, progression and premature mortality. However, telomere shortening can be counteracted by activity of the cellular enzyme telomerase [45]. Telomerase adds telomeric repeat sequences to the ends of chromosomal DNA, thus preserving telomere length, cellular function, and long-term immune function [46]. The introduction of the hTERT gene is a widely used strategy to extend the lifespan of many cell types and successful immortalization has been reported in human retinal pigment epithelial cells, porcine umbilical vein endothelial cells and cattle type II alveolar epithelial cells [27]–[29]. In the present study, primary porcine small intestinal epithelial cells were transfected with the pCI-hTERT-neo plasmid and stable transfectants were selected with G418. The newly established epithelial cell line (ZYM-SIEC02) was detected to have a significantly higher expression of hTERT and higher telomerase activity compared to primary cells, suggesting that the lifespan of pSIECs was successfully extended.

Various synthetic culture media, supplemented with 5–20% FBS, have been used in studies of the mammalian intestinal epithelium [47], And supplementation with ITS or EGF is beneficial for epithelial cell proliferation [2]. In the present study, the addition of 5% FBS was found to be optimal for the growth of ZYM-SIEC02 cells. ITS supplementation promoted cell proliferation, while the combination of ITS and FBS further enhanced proliferation. In contrast, EGF had minimal effects on the proliferation of ZYM-SIEC02 cells. Overall, our data suggests that ITS can partially substitute for the mitogenic effects of FBS and is capable of sustaining proliferation of ZYM-SIEC02 cells, which is beneficial for future studies on viral infection.

The newly established ZYM-SIEC02 cell line was not transformed and was not tumorigenic in nude mice, indicating that this cell line can be used in addition to pSIECs. Furthermore, we found that this cell line closely mimics the porcine intestinal environment *in vivo,* contributing to the establishment of a porcine-derived infection model.

The functionality of the intestinal epithelium is critically important to neonatal swine as this period of time represents a window of significant vulnerability to pathogens including *Salmonella enteria serovar typhimurium (S. Typhimurium)* [48]. Moreover, we detected the invasion efficiency of *S. Typhimurium* in the ZYM-SIEC02 cells and found *S. Typhimurium* could invade host cells effectively and we used MOI of 100∶1 (*S. Typhimurium*: cell) to ensure enough number of intracellular bacteria.

Cytokines impact on the functional state of immune cell populations by eliciting altered patterns of gene expression [2]. In response to pathogenic microorganism infection, host cells secrete numerous types of cytokines and cytokine expression patterns are species- and tissue-dependent [2]. Melania etal [49] found that all porcine intestinal sections tested were able to sense *S. Typhimurium* presence as indicated by changes in IL8 and TNF-α gene expression. Our study of IL8 and TNF-α gene expression suggests that this newly established porcine intestinal epithelial cell line could respond similarly to bacterial infection, we also showed that the ZYM-SIEC02 cells increased secretion of IL-8 infection by *S. Typhimurium.* This is in agreement with previous observations showing increased levels of IL-8 production in the serum of *S. Typhimurium* infected piglets [49], and is also in accordance with a marked upregulation of IL-8 secretion observed in human intestinal epithelial cells and IPEC-J2 cells following *S. Typhimurium* infection [2], [50]. These results suggest that the ZYM-SIEC02 cell line can effectively respond to pathogen infections and that an inflammatory process has been triggered along after infection with *S. Typhimurium* Interestingly, while previous studies demonstrated that IPEC-J2 was of hyporesponsiveness to LPS with respect to IL-8 mRNA expression and secretion [32]. This is consistent with our results but in contrast to the conclusions drawn from microarray analysis following stimulation with 1 µg/ml LPS [51].

Previous work has revealed conflicting results regarding changes in TNF-α gene and protein expression. Several groups observed increase in TNF-α, IL-8 protein and mRNA expression in intestinal tissues from pigs infected with *S. Typhimurium* [52], [53], however, Hyland et al [54] reported that neither TNF-α mRNA nor protein was detected in pigs that infected with Salmonella ssp. In this study, *S. Typhimurium* elicited a modest increase (*P*<0.05) in TNF-α gene expression from ZYM-SIEC02 cells compared to untreated wells. Furthermore, TNF-α mRNA levels were significantly decreased after LPS stimulation, and indicating that TNF-α may not be critical in the early response to LPS as it is in IPEC-J2. We did not detect any TNF-α production from ZYM-SIEC02 cell challenged with *S. Typhimurium* or LPS. TLR4 was upregulated following stimulation with LPS, as the expected values, due to the function of TLR4 as the LPS receptor. The observation that LPS can be the stimulus for IL-6 mRNA production by ZYM-SIEC02 cells suggests that TLR4 were involved in the activation of porcine intestinal epithelial cells in response to LPS. Interestingly, we reported here that ZYM-SIEC02 cells express mRNA for TLR4 and TLR6 and were upregulated significantly after stimulation with LPS.

TLR4 signaling is known that two major signaling pathways, the MyD88-dependent and TIR-domain-containing adapter-inducing interferon-β (TRIF)-dependent signaling pathways, are activated when TLR4 recognizes LPS. Previous studies have demonstrated that the induction of postnatal tolerance in mouse and human small intestine is a mechanism that relies on microRNA (miR)-146a upregulation and subsequent IRAK-1 degradation [55]. Basing on this point, it is also reasonable for TLR4 expression as IRAK-1 is an adapter molecule for MyD88-dependent signaling pathway. The intracellular recognition of LPS initiates the TRIF-dependent pathway, which is important for the induction of adaptive immune responses. It is possible that strategies aimed at opening the TRIF-dependent pathway or others will broaden therapeutic opportunities for controlling TLR trafficking and localization. This may expain why both TLR4 and TLR6 were increased. Be that as it may, the results of our studies establish a physiologically significantly role played by TLR-4 in mediating signaling elicited by LPS. Whether the intracellular recognition of LPS may initiate TRIF-dependent pathway is currently being investigated.

In summary, the newly established ZYM-SIEC02 cell line retains the morphological and functional features of primary swine intestinal epithelial cells. Moreover, this cell line is not transformed and can be safely used for future studies. Therefore, the establishment of a stable ZYM-SIEC02 cell line is of great importance for future studies of the mechanisms of pathogen infection *in vitro*, particularly for swine based infection studies, and also a potential model of zoonotic infections (e.g. *S. Typhimurium*). Given the high degree of homology between porcine and human intestines, studies performed on the porcine intestinal epithelial ZYM-SIEC02 cell line may provide valuable insight into human intestinal disease.
